# Describing water intake in six countries: results of Liq.In^7^ surveys, 2015–2018

**DOI:** 10.1007/s00394-018-1746-6

**Published:** 2018-06-15

**Authors:** Jodi D. Stookey, Jürgen König

**Affiliations:** 1 Children’s Hospital Oakland Research Institute, Oakland, CA USA; 2 Department of Nutritional Sciences, University of Vienna, Vienna, Austria; 3 San Francisco, USA

## Introduction

In 2015, a collection of papers in this journal reported Liq.In^7^ survey findings, which suggested that, in many countries around the world, water intake may not meet adequate intake (AI) recommendations [1]. The papers signaled variability in water intake by sex, socio-economic status, and region. The papers called for further work to standardize methodologies for water intake assessment across countries and deepen understanding of water intake inadequacy and variability. This supplement provides an update about work undertaken since 2015 in these areas.

## Liq.In^7^ status in 2015

In 2015, Liq.In^7^ survey data indicated that over 20% of children, 60% of men, and 40% of women in the populations studied in 12 countries did not meet AI standards for water defined by the European Food Safety Authority (EFSA) [2, 3]. The data motivated research to confirm these prevalence estimates and follow-up regarding possible correlates and consequences, because if valid and generalizable, such high prevalence estimates could signal huge potential for worldwide public health problems related to suboptimal water intake. In the short term, acute water deficit of 2% of body weight or more alters mood, increases cardiovascular strain and fatigue, and decreases alertness, endurance performance, and work capacity [4, 5]. Dehydration is associated with heat stress, reduced work safety and productivity [6], lost wages, and expenses for hospitalization due to acute injury or sickness [7]. If sustained over the longer term, chronic poor hydration is associated with increased risk of chronic disease incidence, progression and/or complications, and mortality [8]. Dehydration significantly magnifies the risk of dying within a year for older adults hospitalized for respiratory illness, gastroenteritis, other gastrointestinal conditions, urinary system infections, cancer, sepsis, cardiac diagnoses, frailty, diabetes and other metabolic disorders [9].

In 2015, Liq.In^7^ survey data from 13 countries suggested potential for water intake disparities between countries. The estimated proportion of survey participants who did not meet the EFSA AI recommendation for water ranged by 80 percentage points among children and adolescents (from 10% in Uruguay to > 90% in Belgium) [2] and by about 40 percentage points among adult men and women (between 20–30% in Germany and 60–80% in Japan) [3].

Dehydration due to insufficient water is considered a “potentially preventable” condition. Expenses for emergency department visits and hospital admissions attributable to dehydration are classified as “potentially preventable spending” and indication of need for quality improvement in community-level systems [10]. It is assumed that dehydration and its sequelae can be avoided if effective ambulatory care services, community-level interventions and/or social measures are in place [11, 12]. Because dehydration is avoidable, between-group differences in water intake sufficiency qualify as health inequities (“*avoidable* inequalities in health between groups of people” [13]), which were declared to be politically, socially, and economically unacceptable by Heads of Government, Ministers and government assembled by the World Health Organization [14].

“Ensuring access to safe drinking water and sanitation for all members of the population, without discrimination, is an obligation for all governments. Everybody, whether rich or poor, men, women and children, people living in urban and rural areas, having a suitable accommodation or not, people with physical disabilities or people living in institutions such as prisons or hospitals, has the right to access these services [15].”

## Liq.In^7^ progress relative to what benchmark?

Tables 1 and 2 summarize the progress of Liq.In^7^ projects over the past 3 years relative to global health priorities and best practices for public health intervention. The tables map contributions of the Liq.In^7^ papers in this supplement to dimensions of water intake that are prioritized by the United Nations (UN) [16], as well as domains for data collection recommended by the WHO Commission on Social Determinants of Health (SDH) [17] and US Centers for Disease Control [18]. Table 1 focuses on community-level determinants of adequate water intake. Table 2 focuses on individual-level determinants of adequate water intake.

**Table 1:** Recent Liq.In^7^ data analyses by community-level determinants of adequate water intake

Domains for community-level data prioritized by Health Authorities			Determinants of adequate water intake	References for Liq.In^7^ data analyses 2015–2018					
UN	WHO	CDC		Argentina	Brazil	China	Indonesia	Mexico	Uruguay
Water availability	1, 2	Environment	Country	[24–26]	[24–26]	[26]	[26]	[24–26]	[24–26]
			Within-country region, State			[27]	[28]		
			City						
			Within-city locations, neighborhood	[29]	[29]	[29]	[29]	[29]	[29]
		Social	Social norms	[29]	[29]	[29]	[29]	[29]	[29]
			Water policies						
Water acceptability	1, 2	Environment	Water quality, perception, color, odor, taste						
			Water facilities are culturally sensitive						
		Education	Media, communications about water						
			Health education curricula, training systems						
		Social	Social networks (e.g. household type, peers)						
			Organizations, champions for drinking water						
Water Affordability	1	Economic	Water price, affordability						
			Income, poverty, income disparity			[27]	[28]		
			Market forces						
			Water financing						
Water accessibility	2	Environment	Rainfall, drought						
			Water availability at school/work	[29]	[29]	[29]	[29]	[29]	[29]
			Water availability at home, housing quality	[29]	[29]	[29]	[29]	[29]	[29]
			Water availability in public spaces, libraries	[29]	[29]	[29]	[29]	[29]	[29]
			Water availability in food retail, businesses						
			Types of fluid available, composition	[24–26, 29]	[24–26, 29]	[26, 29]	[26, 29]	[24–26, 29]	[24–26, 29]
			Amount, quality of food available	[29]	[29]	[29]	[29]	[29]	[29]
		Health care	Health care services (e.g. doctor, nurse advice)						
Water safety	2	Environment	Free from micro-organisms, contaminants						
			Barriers to accessing water (e.g. area safety)						
Water sufficiency	2	Environment	Altitude, climate, temperature, humidity						
	5	Economic	School/work absences, loss of earning, costs						

*UN* The United Nations describes the human right to water in terms of water availability, acceptability, affordability, accessibility, safety and sufficiency [16]. *WHO* The World Health Organization recommends five levels of community-level data collection (level 1: society, level 2: environment, level 3: population group vulnerability, level 4: individual treatment/access to care, level 5: consequences of poor health outcome) [17]. *CDC* The United States Centers for Disease Control recommends monitoring five key types of social determinants of health (economic, education, social, health care services, neighborhood and build environment) [18]

**Table 2:** Recent Liq.In^7^ data analyses by individual-level determinants of adequate water intake

Domains for individual-level data prioritized by Health Authorities			Determinants of adequate water intake	References for Liq.In^7^ data analyses 2015–2018					
UN	WHO	CDC		Argentina	Brazil	China	Indonesia	Mexico	Uruguay
Water accessibility	3	Environment	Limited access (e.g. disabled, institutionalized, homeless, traveler)						
		Health care	Individual utilization of health care services						
Water affordability	3	Economic	Individual socio-economic status, occupation, education income						
Water sufficiency	3	Behavioral	Type, intensity, duration of physical activity	[26]	[26]	[26]	[26]	[26]	[26]
			Smoking						
			Food						
			Beverage composition						
		Biological	Genes						
			Life course, medical history (insult accumulation, fetal program)						
			Body size	[26]	[26]	[26]	[26]	[26]	[26]
			Age	[24, 25]	[24, 25]	[27]	[28]	[24, 25]	[24, 25]
			Sex	[24, 25]	[24, 25]	[27]	[28]	[24, 25]	[24, 25]
			Thirst						
			Regulation (including circadian)						
			Kidney function, urine volume						
			Skin health						
			Health status, Obesity, Acute/chronic illness						
			Individual requirements overall, combined	[24, 25]	[24, 25]	[27]	[28]	[24, 25]	[24, 25]
	5	Economic	School/work absences, loss of earning, costs						

*UN* The United Nations describes the human right to water in terms of water availability, acceptability, affordability, accessibility, safety and sufficiency [16]. *WHO* The World Health Organization recommends five levels of community-level data collection (level 1: society, level 2: environment, level 3: population group vulnerability, level 4: individual treatment/access to care, level 5: consequences of poor health outcome) [17]. *CDC*: The United States Centers for Disease Control recommends monitoring five key types of social determinants of health (economic, education, social, health care services, neighborhood and build environment) [18]. The Institute of Medicine [22] identifies individual-level behavioral and biological determinants of water intake and water requirements

In 2010, the UN General Assembly and Human Rights Council recognized safe drinking water as a human right, and key to having healthy populations achieve the international Millennium Development Goals [16]. The human right to water was defined in terms of sufficient, safe, acceptable, physically accessible, and affordable water for personal and domestic uses [16].

Best practice to intervene against health disparities is to address “upstream”, community-level or “root causes” of health disparities [17, 18]. Gathering data to identify the social determinants that drive variability in water intake between and within communities around the world is a critical first step towards enabling the kinds of essential public health services recommended by the CDC [18] and tailoring interventions to effectively and sustainably improve the availability, acceptability and/or accessibility of safe drinking water.

The WHO and CDC distinguish type of water source, plain drinking water as opposed to food or other beverages, as an important dimension of water intake. Plain drinking water is the source recommended for meeting water requirements without consuming excess sugar or calories [19, 20].

Various community-level factors, including environmental, socio-cultural, and economic conditions, might conceivably contribute to variation between communities in water intake sufficiency, either by influencing water intake and/or altering water requirements (see Table 1). Differences in local weather (rainfall/drought) and sanitation, water purification and water delivery infrastructure may lead to variation in water availability, water safety, and accessibility. Differences in many aspects of the physical environment, which determine body water losses, including ambient temperature, humidity, altitude, transportation infrastructure, availability of acceptable bathroom facilities, workplace settings, area safety, and public spaces for leisure-time physical activity, can lead to differences in water intake requirements across communities. Differences in the food environment, the types of foods available and their protein and salt content, may create differences in water intake requirements, perceived thirst, and water-seeking behavior. The composition of beverages that are available in markets, food retail, restaurants, schools, workplaces, and public places in the community might translate into differences in the volume of fluid consumed, as a function of macro- and/or micronutrient content, pH and/or osmolality on palatability and water absorption rate [21]. Differences in socio-cultural norms, about where, when, what, how, with whom, and why to prepare and consume fluid, may explain variation in the amount and/or type of fluid consumed. In some communities, a high cost of living may reduce the affordability and intake of drinking water. Between-community differences in physical environment, norms, cost of living, availability, safety, accessibility, affordability and sufficiency of fluids may, in turn, reflect differences in local laws and organizational policies.

Within communities, various individual-level factors might also contribute to variation in water intake adequacy by influencing access to water sources and water intake and/or altering water requirements (see Table 2). Community-level social, economic, and/or physical environments are believed to interact with individual behaviors and biological risk factors to determine an individual’s health [22]. An individual with low socio-economic status might, for example, not be able to afford water and/or have a limited range of accessible options for water sources, reducing the volume and quality of water consumed. At the same time, an individual with low socio-economic status might be more likely to do manual labor outdoors in the heat, instead of clerical work in an indoor air-conditioned office, raising water requirements. Individual water requirements vary by age, sex, body size, diet quantity and quality, physical activity, smoking, past medical history and current health status [5].

## Liq.In^7^ progress 2015–2018

In this supplement, six papers report water intake data that were collected from survey respondents in Argentina, Brazil, Mexico, Uruguay, China and Indonesia, using the same 7-day Liq.In^7^ method, which was validated against water turnover in the US [23]. All six papers focus on beverage sources of water intake, specifically, excluding water intake from food. The papers refer to total “fluid” intake, which includes plain drinking water as well as all other beverages. All six papers describe fluid intake in terms of factors which may be important to inspire deeper investigation into causal mechanisms, inform, educate, and empower people about barriers to water intake adequacy, mobilize community partnerships and tailor interventions to increase water intake (i.e. the 10 Essential Public Health Services [18]). Papers in this supplement take next steps to describe: (1) variation in water intake by community, (2) community-level resources associated with water intake variability, (3) individual-level determinants of water intake variability, (4) water intake adequacy according to AI standards other than EFSA, (5) intake of plain drinking water as part of a whole beverage intake pattern, and (6) water intake relative to food intake.

### Steps taken to describe variation in water intake by community

Gandy et al [24] and Martinez et al [25] describe between-country differences in total fluid volume. For children, ages 6–9 years, surveyed in Argentina, Brazil, Mexico and Uruguay the mean total fluid intake estimates ranged from as low as 1.6 L/d to as high as 1.9 L/d. For adolescents, ages 10–17 years, total fluid intake ranged from 1.7 to 1.9 L/d [24]. For adults in these countries, the mean total fluid intake ranged from 1.7 to 2.3 L/d [25].

Morin et al [26] describe between-country differences in fluid volume and fluid composition or pattern. Whereas Indonesian survey participants reported a high total fluid volume, consisting mostly of drinking water, survey participants in Latin American countries tended to report a low total fluid volume with a high proportion of fluid intake coming from sugar-sweetened beverages.

Zhang et al [27] and Laksmi et al [28] describe within-country, regional variation in total fluid volume. In China, mean total fluid intake was 200 ml/d higher for participants living in the northern region vs the northwestern region [27]. In Indonesia, the mean total fluid intake in the Jabodetabek region was more than 800 ml/d higher than in the West Java region [28].

Morin et al [29] describe within-community differences in the pattern of fluid intake. In all countries studied, sugar-sweetened beverages accounted for 21–44% of beverage intake at school. In all countries studied, drinking water accounted for less than a third of intake at locations other than home, school or workplace. Further work is needed to determine why people drink beverages other than plain water in public places, food retail or restaurants, and what supports schools in these communities might need to reduce sugar-sweetened beverage intake.

Further studies, designed to test for between-country differences in total fluid volume and pattern, with aligned sampling and recruitment strategy and attention to confounding variables, are needed to test hypotheses regarding the magnitude of differences between countries and the community-level resources, policy or infrastructure that mediate variation in fluid intake.

### Steps taken to describe community-level resources associated with water intake variability

Two papers in this supplement describe significant differences in total fluid intake by income level. In China, mean total fluid intake was significantly higher, by approximately 200 ml/d, for survey participants from higher income (Tier 1) cities compared to lower income (Tier 2 and Tier 3) cities [27]. In Indonesia, mean total fluid intake varied by more than 300 ml/d over the range of household income reported by participants [28]. The relationships with income suggest potential for associations between fluid intake and resources associated with income, such as household electricity usage and cooking equipment, water affordability and accessibility.

Morin et al [26] consider community-level resources, such as type of water source (tap, well or spring), availability of drinking water fountains at school, and/or fluids for purchase through vending machines or snack shops at school, as potential determinants of between-country differences in fluid intake patterns.

### Steps taken to describe individual-level determinants of water intake

Morin et al [26] characterize between-country differences in total fluid volume and beverage pattern in terms of variables that alter individual water requirements: weight status, physical activity level and screen time. They associate, for example, the high total fluid intake from a high intake of drinking water, in Indonesia, with underweight status and having a physical activity level of once a week to twice a month.

Total fluid intake did not differ significantly by sex among adolescents or adults in all of the countries studied, except for adults from Brazil [24, 25, 27, 28]. Given, however, that the AI recommendations for adolescents and adults from EFSA, the IOM, China and Indonesia differ by sex, the data suggest need to explore why beverage volume did *not* vary by sex. It is not known if factors such as the size of the container from which fluid is consumed, cultural norms or beliefs about how much is normal to consume, and/or location of consumption limit the volume consumed for males.

### Steps taken to describe water intake sufficiency or adequacy

Four papers in this supplement address uncertainty in the prevalence estimates reported in 2015 related to the use of European standards (the EFSA AI) to assess water intake in non-European countries.

Gandy et al. [24] and Martinez et al. [25] describe total fluid intake adequacy for samples of children, adolescents, and adults from Latin America relative to AI recommendations developed by the IOM for North America [5]. Latin American country-specific AI were not available. As the IOM AI recommendations for adolescents and adults are higher than the corresponding EFSA AI values, the new prevalence estimates are higher for adolescents and adults, by as much as 14 percentage points for adolescents and as much as 31 percentage points for adults [23]. Under- or overestimating the prevalence by such a large amount may have serious implications for public health funding and programs. The discordant estimates underscore need to determine water intake requirements in Mexico, Brazil, Uruguay, and Argentina to identify country-specific AI and narrow the margin of error in estimates of the prevalence of total fluid intake below recommendations.

Laksmi et al. [28] and Zhang et al. [27] describe total fluid intake relative to country-specific AI in Indonesia and China, respectively. Consistent with the EFSA-based analyses [2], the new data continue to suggest that about one quarter of children, adolescents and adults surveyed in Indonesia and over half of children, adolescents, and adults surveyed in China do not meet water intake recommendations. In Indonesia, the prevalence of total fluid intake below the AI tended to be a few percentage points higher when estimated relative to the local Indonesian AI than when estimated relative to the EFSA AI [28]. The data from China suggest that about 50% of children surveyed, ages 4–10 years old, might need to increase water intake by 200–400 ml/d or approximately 1–2 cups of water per day to meet the Chinese AI.

### Steps taken to describe water intake as part of whole beverage pattern

All six papers in this supplement distinguish plain drinking water from other beverage sources of total fluid intake. The data open the door to estimating how much plain drinking water each age- and sex-specific group might need to consume to meet the AI for water intake, without consuming excess calories or added sugar from beverages. The papers also provide preliminary data regarding what specific beverage categories would need to be replaced with drinking water to meet the AI for water intake with plain drinking water. Further work is needed to stratify beverage pattern by whether or not the total fluid intake meets the AI.

The data in this supplement may be useful for planning interventions to change both absolute and relative fluid intake. Across countries and age groups, different caloric beverages are consumed. For children ages 4–9 years in Indonesia and China, for example, milk is the primary caloric beverage, followed by juice [27, 28]. In China, 95% of children consume milk, with half of the consumers drinking 239–388 ml/d [27], and carbonated sweetened drinks account for 3% or less of daily fluid intake [27]. For adolescents in Latin America, carbonated sweetened drinks are the primary caloric beverage [24]. For adults in China and Indonesia, carbonated sweetened drinks account for only 4% of beverages, but milk and sweetened ready-to-drink tea and coffee account for over 15% of beverage intake [27, 28]. In Mexico, 82% of adults consume more than one serving of sugar-sweetened beverages daily [25]. Together, the data signal that drinking water interventions aiming to increase intake of plain drinking water and decrease intake of caloric beverages should be tailored to the location- and group-specific pattern of caloric beverage intake.

### Steps taken to describe water intake relative to food

Morin et al. [29] describe the pairing of drinking water and other beverages with food for six countries. In Indonesia, consistent with the high intake of drinking water at home, drinking water accounts for 84% of fluid consumed at meals. In contrast, in Brazil, Uruguay, Argentina, Mexico, and China, other beverages account for the majority of fluid consumed with meals. In these five countries, respondents report consuming drinking water *without food*. Studies are needed to understand cultural beliefs and expectations regarding what drink(s) to pair with food(s).

Morin et al. [29] consider one aspect of food preparation, frequency of parents preparing a lunchbox for their child, as a potential determinant of fluid intake variability in children. They observe that having a lunch box prepared daily distinguished the fluid intake pattern in China from the patterns in other countries. Conversely, not having a lunch box prepared daily distinguished beverage intake patterns in Latin American countries.

## Summary of progress and directions for the future

Tables 1 and 2 summarize how recent Liq.In^7^ data analyses contribute to the knowledge base about water intake in six countries and point to gaps in knowledge that remain for future research. The new Liq.In^7^ descriptive analyses address questions about water availability and sufficiency in six countries for healthy children, adolescents, and adults, and prompt deeper, hypothesis-driven work to confirm and characterize findings.

**Fig. 1:**
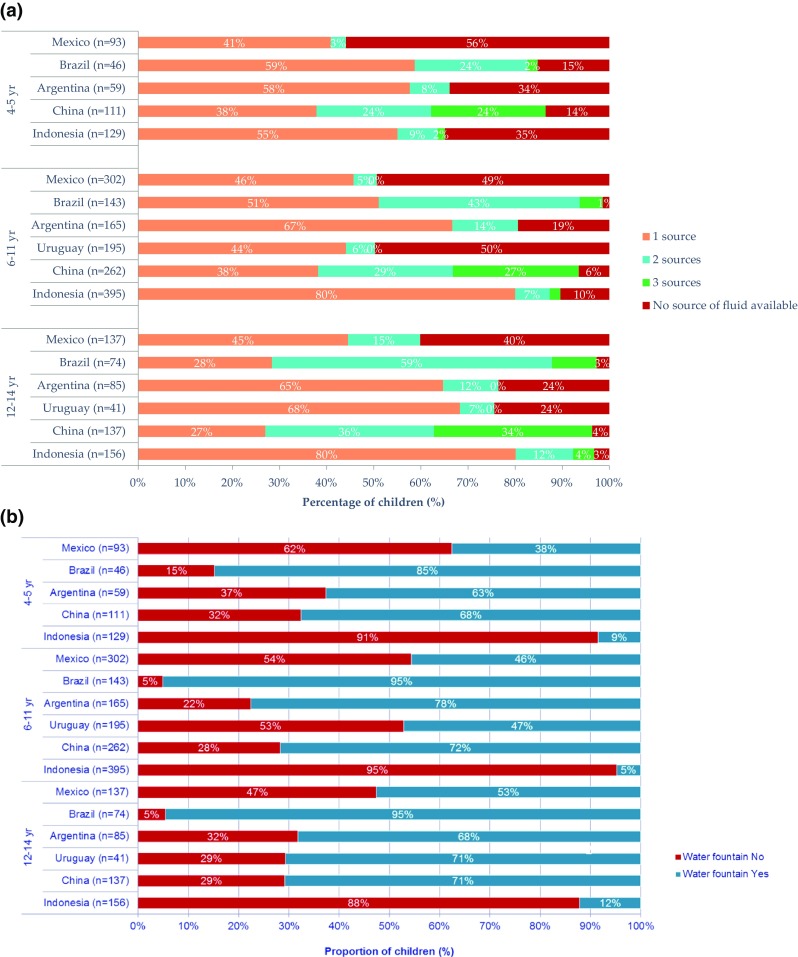
**a** Number of sources of fluid intake reported by parents to be available at school by child age and country. **b** Availability of a drinking water fountain at school by child age and country. Liq.In^7^ survey respondents in each country were asked the same yes or no question about drinking water fountains, ‘Do you have a water fountain available at school?’, and vending machines, ‘Do you have a vending machine available at school?’. Respondents in Brazil, China and Indonesia were also asked ‘Do you have a grocery or school canteen available at school?’

Many potential determinants of water intake disparities, including water safety, acceptability, accessibility, affordability, social norms and policies, remain to be explored for healthy individuals as well as vulnerable population groups, in each country. The Liq.In^7^ surveys are in position to address some of the remaining gaps in the next few years through analysis of data that have already been collected (e.g. systematic analysis of water intake timing and location) or through collection of new data. The standardized Liq.In^7^ survey protocol and periodicity make it possible to add survey elements. In 2016, for example, in addition to completing a 7-day fluid record, parents also answered new questions about the types of fluid available at the children’s schools. The data collected in Mexico, Brazil, Argentina, China and Indonesia suggest marked differences in school availability of fluid for children ages 4–9 years (see Fig. 1a, b) [Personal correspondence, Clementine Morin & Isabelle Guelinckx, 2018]. Unpublished 2016 data, furthermore, suggest that the dependence of children on sources of water intake at school may also vary by country, because 45, 52, 64, 85, and 92% of parent respondents in Mexico, China, Indonesia, Brazil, and Argentina report that they do *not* send their child to school each day with a lunch box.

Over time, data accumulating from Liq.In^7^ surveys may enable trend analyses, age, period, cohort analyses, and population-based quality improvement projects and program evaluations. Finally, the Liq.In^7^ survey data provide key preliminary data to inspire and justify research by anthropologists, psychologists, sociologists, economists, and public health policymakers on determinants of water intake disparities.
